# Prone versus Supine FDG PET/CT in the Staging of Breast Cancer

**DOI:** 10.3390/diagnostics13030367

**Published:** 2023-01-19

**Authors:** Lara Nassar, Mutaz Kassas, Alain S. Abi-Ghanem, Malak El-Jebai, Safaa Al-Zakleet, Amro S. Baassiri, Rami Abou Naccoul, Andrew Barakat, Arafat Tfayli, Hazem Assi, Ghina Berjawi, Enrique Estrada-Lobato, Francesco Giammarile, Sobhan Vinjamuri, Mohamad Haidar

**Affiliations:** 1 Department of Diagnostic Radiology, American University of Beirut Medical Center, Beirut 1107-2020, Lebanon; 2 Yale Cancer Center, Yale School of Medicine, New Haven, CT 06520-8028, USA; 3 Department of Internal Medicine, Division of Hematology and Oncology, Naef K. Basile Cancer Institute, American University of Beirut Medical Center, Beirut 1107-2020, Lebanon; 4 Nuclear Medicine and Diagnostic Imaging Section, Division of Human Health, International Atomic Energy Agency, Vienna 1400, Austria; 5 Nuclear Medicine, Royal Liverpool and Broadgreen University Hospital, Liverpool L7-8YE, UK

**Keywords:** FDG PET/CT, prone acquisition, breast cancer, nuclear, staging

## Abstract

Supine [18F]Fluorodeoxyglucose (FDG) positron emission technology/computed tomography (PET/CT) is a commonly used modality for the initial staging of breast cancer, and several previous studies have shown superior sensitivity and specificity of prone FDG PET/CT in comparison to its supine counterpart. This retrospective study included 25 females with breast cancer referred for staging. They underwent supine FDG PET/CT followed by prone FDG PET/CT. The outcomes were: number of primary breast lesions, anatomical site of FDG-avid lymph nodes (LNs), and number and type of bone lesions, with SUVmax of all corresponding parameters. Performance was superior in prone acquisition compared to supine acquisition, with the respective results: 29 vs. 22 breast tumor lesions detected, 62 vs. 27 FDG-avid axillary LNs detected, sensitivity of 68% vs. 57%, specificity of 64% vs. 53%. The detection rate of axillary LNs in the prone position was significantly higher (*p* = 0.001). SUVmax for breast tumor lesions (*p* = 0.000) and number of detected axillary LNs (*p* = 0.002) were significantly higher in prone acquisition. Five patients were upstaged after experts read the prone acquisition. Prone FDG PET/CT acquisition is a promising technique in detecting primary breast lesions and metastatic LNs possibly missed in supine acquisition, which may lead to change in patient staging and management.

## 1. Introduction

Breast cancer continues to be the most-diagnosed cancer worldwide, with an increase in incidence rates in the United States during recent years [1,2]. As a consequence of aging and population growth, the breast cancer burden will continue to grow, with over 3 million new cases expected by year 2040 [1]. According to the National Comprehensive Cancer Network (NCCN) guidelines on the workup of patients with breast cancer, [18F]Fluorodeoxyglucose (FDG) positron emission tomography/computed tomography (PET/CT) can aid in the identification of unsuspected regional nodal disease and/or distant metastasis in locally advanced breast cancer [3]. The current available literature shows that the use of FDG PET/CT may lead to significant change in the staging and choice of treatment in patients with recently diagnosed breast cancer [4]. A study by Groheux et al. [5] reinforces the NCCN statement regarding the increasing ability of FDG PET/CT to modify patient staging when the breast cancer proves to be more aggressive. A review conducted by Nicole et al. [6] found that FDG PET/CT has high specificity and positive predictive value ranges (80–100% and 50–100%, respectively) for axillary staging and a sensitivity of 91% in the detection of tumors larger than 1 cm. Vogsen et al. [7] found that FDG PET/CT is also advantageous for the detection of local or distant recurrence with a sensitivity and specificity of 88% and 98%, respectively. Nonetheless, there are also some pitfalls when using FDG PET/CT: one prospective study by Wahl et al. [8] demonstrated that FDG PET/CT in breast cancer is likely to have false negatives when staging the axillae in the case of fewer and smaller tumor-involved lymph nodes (LNs). Moreover, physiologic uptake observed in benign processes and inflammatory changes may show FDG avidity [9]. Accordingly, higher sensitivity is necessary in axillary staging and detecting primary breast tumors smaller than 1 cm in order to maximize the utility of FDG PET/CT.

One possible approach is the modification of positioning during imaging. Prone position acquisition has been shown to be favorable over supine position because it allows for superior separation of deep breast structures from the chest wall and better relaxation of the pectoral muscles, which may allow for better visualization of LNs for staging [10]. The objective of this study is to compare the sensitivity and specificity of supine and prone breast FDG PET/CT scans for breast cancer patients, especially for small lesions and LNs, while denoting any subsequent changes in management.

## 2. Materials and Methods

### 2.1. Patient Selection

The Institutional Review Board at the American University of Beirut approved this retrospective study, which includes 29 female patients with biopsy-proven breast cancer referred for whole body time-of-flight (TOF) FDG PET/CT. These patients presented to the Nuclear Medicine Division at the American University of Beirut Medical Center (AUBMC) between January 2015 and November 2015. Four patients presenting for re-staging were excluded, which rendered the number of analyzed patients to 25 (n = 25) in total.

### 2.2. Imaging Technique

Patients were instructed to fast for at least 6 h before the injection of FDG. Blood glucose levels were measured before injection, and the injected activity ranged between 185–296 MBq. After injection, patients were kept lying comfortably. The whole-body FDG PET/CT in supine position was performed approximately 60 min after injection using a Philips Gemini TF 16 PET CT. The time per frame of PET scan was 2 min/frame for all patients in supine and prone acquisition. The CT images were first acquired, followed by the PET study. After completion of the PET acquisition, images were attenuation-corrected, then fused with the CT images. This protocol was applied for both prone and supine position acquisitions. After supine acquisition, patients were then positioned in prone for breast PET/CT acquisition. This was performed 10 min at most after completion of supine acquisition in order to minimize time differences, which may influence image parameters such as SUVmax and tumor-to-background ratios. During prone acquisition, the patient’s breasts were positioned in a custom-built mattress made of poly foam and plexi-glass covered with leather, designed and produced at AUBMC, and the patients’ arms were elevated above the head. Images were reconstructed to a high resolution of 2 mm slice thickness.

### 2.3. Image Interpretation

Two board-certified nuclear medicine physicians with more than 10 years of experience in nuclear medicine interpreted the FDG PET/CT images. Both positions were interpreted separately and the physicians were blinded to each other. The processing and viewing of images were attained using a semi-quantitative workstation. Image interpretation was performed with IntelliSpace Portal 8.0 by Philips Healthcare, and images were simultaneously displayed as PET, CT, and PET/CT fusion series in coronal, sagittal, and axial cuts, as well as 3D maximum-intensity projections (MIP). Images were assessed for primary breast lesion size and their corresponding average SUVmax, presence or absence of LNs and their respective average SUVmax, and presence or absence of osteolytic/osteoblastic bone lesions noting their anatomical site and SUVmax if present. Lesions were measured in centimeters (cm) on CT imaging. Breast lesions and LNs showing an SUVmax equal to or greater than 2 were considered positive. Patients were staged according to the American Joint Committee (AJCC) on Cancer 8th Edition for Breast Cancer Staging [11].

### 2.4. Statistical Analysis

**Figure 1:**
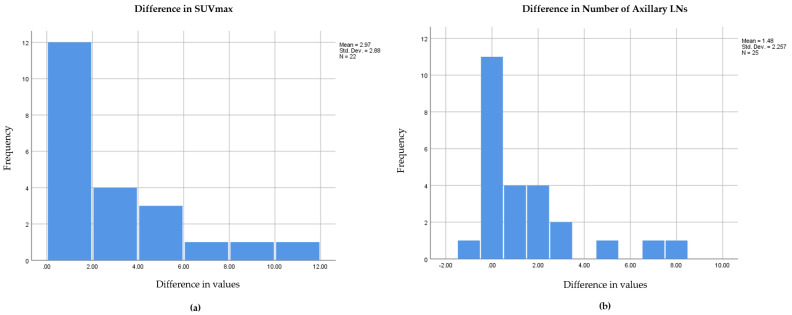
Histograms of difference in SUVmax (**a**) and difference in number of axillary LNs (**b**) showing skewed distribution.

Statistical analysis was performed on the SPSS software (release 23.0; SPSS Inc., Armonk, NY, USA) for Windows. Measurements were made in centimeters, and quantitative data was described with mean and range (minimum–maximum). The difference in axillary LN detection between prone and supine positions was tested using Fisher’s exact test. The difference in the following variables of imaging between supine and prone acquisition were also examined: SUVmax of primary breast lesions and number of detected axillary LNs. The paired t test was preferred; however, the difference in means was not normally distributed for both variables as seen in Figure 1, so a paired Wilcoxon test was used. Results were considered statistically significant at a *p*-value < 0.05 (two-sided).

## 3. Results

### 3.1. Primary Breast Lesions

In the prone position, 29 breast tumor lesions ranging from 0.30 cm up to 4.70 cm were detected. The mean SUVmax was 9.44 (range: 2.30–32.90). Three of the twenty-nine lesions were smaller than 0.5 cm. In the supine position, 22 breast tumor lesions ranging from 1.10 cm up to 4.7 cm were detected. The mean SUVmax was 7.33 (range: 2.10–30.20). Both position acquisitions detected all the multiple breast lesions in one patient out of 25, presented in Table 1.

Differences between supine and prone acquisition demonstrated the following

**Figure 2:**
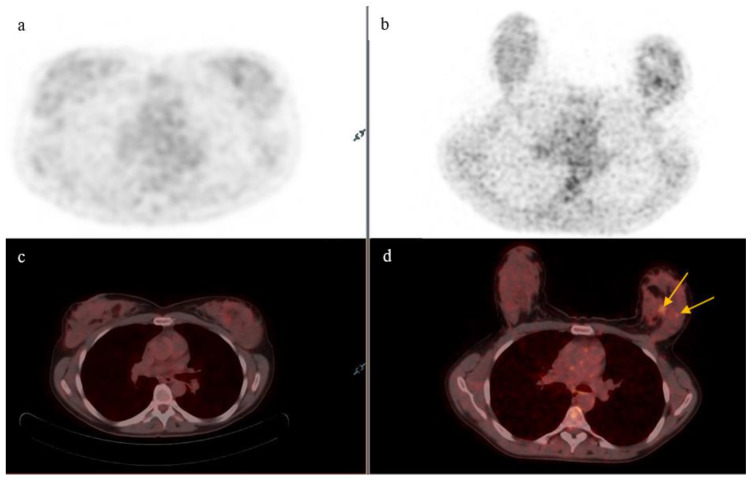
Axial PET and fused PET/CT images of the breast. Two small lesions (yellow arrows) are visualized within the left breast in prone acquisition (**b**,**d**), corresponding to multi-focal disease. Supine acquisition (**a**,**c**) showing single focal uptake in the left breast.

- On supine position acquisition, primary breast lesions were missed in three patients, all of which were detected on prone position. One such example is seen in Figure 2,
- A single lesion was detected on supine position in two patients who, on prone position, each had two lesions detected (Table 1),
- One lesion was detected on supine position in a patient who was found to have three lesions on prone acquisition (Table 1).

### 3.2. Lymph Nodes

**Figure 3:**
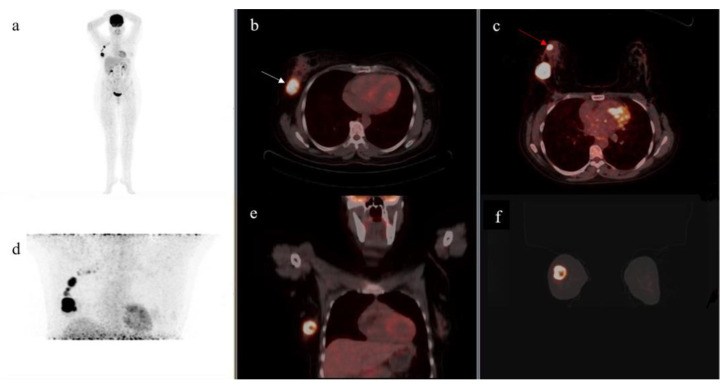
MIP (**a**,**d**) showing FDG avid lesions in the right breast with axillary lymph node metastasis. Fused axial PET/CT images in supine (**b**) showing a lesion (white arrow) in the right breast, which is better delineated on prone (**c**) with the visualization of an additional lesion (red arrow). Fused coronal PET/CT cuts of supine (**e**) and prone (**f**) acquisition are displayed.

FDG-avid axillary LNs were detected in 15 patients on prone acquisition; however, supine acquisition detected FDG-avid axillary LNs in 10 patients. One example of a patient with a secondary breast lesion detected in prone is presented in Figure 3. On prone acquisition, a total of 62 FDG-avid LNs were detected with mean SUVmax of 5.53 (range: 2.10–16.48). On supine acquisition, a total of 27 LNs were detected with average SUVmax of 6.13 (range: 3.30–15.00). One patient had 12 FDG-avid nodes on prone with only 4 FDG-avid nodes showing on supine. Concerning the five patients whose lesions were missed on supine imaging, prone imaging had detected a single node in two patients, two nodes in two other patients, and three nodes in the last patient; their SUVmax range was 2.10–3.10, presented in Table 2.

**Figure 4:**
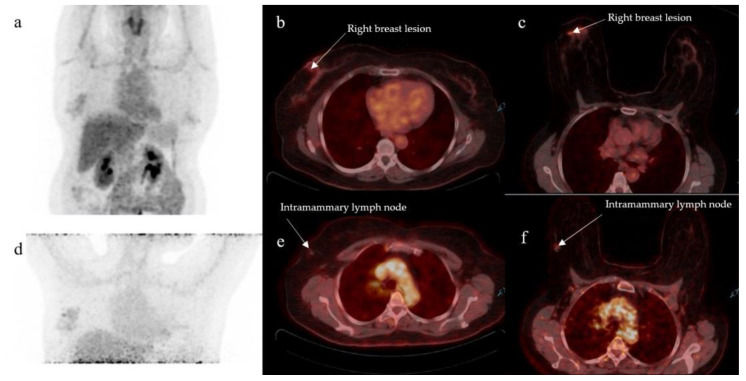
MIP images (**a**,**d**) showing uptake in the right breast. Fused axial PET/CT cuts showing faintly FDG-avid on supine right breast lesion (**b**), which is better visualized on prone (**c**). An intramammary lymph node appears non-radiotracer avid on supine (**e**) while it appears avid on prone (**f**).

In the prone position, a single intramammary LN was detected in two patients with SUVmax of 1.60 and 3.20; neither LN was detected in supine position, and one patient is presented in Figure 4. Both positions detected a total of three internal mammary FDG-avid nodes in two patients. Their SUVmax on prone vs. supine were 7.06 vs. 4.90 and 3.90 vs. 3.60, respectively. Prone position was able to detect supraclavicular FDG-avid nodes in two patients, one of which was missed on supine. The SUVmax for the missed FDG-avid node was 2.06 on prone. No mediastinal FDG-avid nodes were detected by either acquisition technique.

### 3.3. Bone Metastasis

Both positions equally detected bone lesions in the same two patients. Notably, the lesions were osteolytic in nature.

### 3.4. Pathology

Pathology biopsy reports indicating the type of primary breast tumor were available for all patients. Surgical reports detailing the pathology of 47 axillary LNs out of the total 62 identified via prone were also reviewed. Rates for prone vs. supine sensitivity were 68% vs. 57% and rates for prone vs. supine specificity were 64% vs. 53%, respectively. Axillary LN metastases were missed on both acquisitions in two patients where three LNs were positive in one case with eight positive LNs in the other. In one other patient, 8 vs. 3 LNs were detected on prone and supine acquisitions, respectively, where pathology came back positive for 11 LNs. Concerning false positives, three axillary LNs were detected in one patient on both acquisitions, while the pathology report came back negative for malignancy.

Using Fisher’s exact test, the higher detection rate of axillary LNs on prone position acquisition was significant (*p* = 0.001). The paired Wilcoxon test found a significant difference in the SUVmax between prone and supine positions (*p* = 0.000) and number of axillary LNs between both positions (*p* = 0.002).

### 3.5. Staging and Prognosis

There was a change in patient staging in 5/25 patients (20%): all 5 patients were upstaged, with no downstaging in any patient. In one patient where supine acquisition was only able to detect axillary LNs, prone acquisition was able to detect one additional supraclavicular LN. This additional LN was considered a true positive because a FDG PET/CT acquired following neoadjuvant chemotherapy demonstrated that it was no longer FDG-avid. This patient was stage IIA on supine but was upstaged to stage IIIC on prone. In the rest of the patients, no FDG-avid LNs were detected on supine acquisition, while prone acquisition detected one axillary LN in two patients and two axillary LNs in another two patients. As per postoperative pathology reports, all LNs were confirmed true positives. These five patients had a change in management since neoadjuvant chemotherapy was indicated. To note, both nuclear medicine physicians concordantly upstaged the aforementioned five patients, without downstaging any patient.

## 4. Discussion

Many studies have previously explored the difference between supine and prone FDG PET/CT acquisition and the added benefit of prone positioning when staging patients with breast cancer [12,13,14]. It has been suggested that the breast positioning pads in the prone position immobilize the breasts, which reduces breathing motion artefacts [15]. However, the pads should allow the breasts to hang freely in such a way that the mammary glands are not compressed. Agglomerated LNs that were lumped together were visualized as one large LN on supine images, while prone images allowed for clearer distinction. Although the purpose of our study was not to compare appearances and patterns of uptake on prone versus supine positions, we did notice that heterogeneous rim uptake, faint sub-centimetric uptake, photopenic center, and central necrosis were some lesion characteristics that were appreciated better on prone-acquired images.

As for the materials, the pad should be radiolucent so that it does not result in an artefact of its own. The AUBMC custom-built mattress is made of poly foam and plexi-glass covered with leather, free from metallic material. The leather and plastic plexi-glass, which has been previously reported to be radiolucent, did not cause any artefacts [16]. These conditions improve the qualitative assessment of breast tissue. It is worth noting that some of the patients who underwent prone-positioned PET/CT preferred that position because it was similar to the breast MRI imaging position, which they were already familiar with. Moreover, prone PET/CT would allow for better cross-image comparison with prone MRI and longitudinal image registration for future prone breast PET/CT in case of future or previous imaging [17]. As for the technologists, they denied any position preference.

In our study, prone images detected more breast lesions and displayed a higher SUVmax. Error in attenuation–correction may be considered as a cause for the higher SUVmax. Patients underwent PET/CT in both supine and prone positions, and attenuation correction was performed for both acquisitions. This means that the custom-built mattress used in prone positioning was attenuation-corrected, and it likely did not lead to under or over-estimation of FDG uptake. One other way to explain the higher SUVmax in prone imaging would be due to the delayed acquisition. In this study, supine imaging was performed approximately 60 min after FDG injection, followed by prone acquisition. When considering additional time, 20 min for supine acquisition in addition to 10 min to switch the patient to prone positioning would amount to a 90 min interval from FDG injection until prone acquisition. The European Association of Nuclear Medicine recommends a 60 min interval between injection and start of acquisition [18]. Although there is no clear consensus on the effect of time interval on FDG uptake, one tumor kinetic model by Wangerin et al. [19] has shown increased tumor detectability at 2 h compared to 1 h. There are several studies in the literature comparing prone and supine positioning in breast FDG PET/CT, and their findings shed insight on this possible error. One study on 18 patients by Kaida et al. [13] with a study design of supine FDG PET/CT imaging followed by prone imaging found that prone imaging had a significantly higher sensitivity, accuracy, and primary tumor multifocality than supine imaging. Another study by Kaida et al. [20] with similar study design, where prone acquisition was performed after 86 min, revealed that two patients with malignant breast lesions missed on supine were apparent on prone acquisition. This mirrors a finding in our study, where three patients with unique breast tumor lesions were detected on prone acquisition but missed on supine. It may be argued that these studies, which have similar study design and study findings to our own, suffer from an error in the study design leading to delayed acquisition and ultimately falsely higher detectability. However, Erdogan et al. [21], who also had a similar study design, failed to show any significant benefit for the evaluation of local disease. Moreover, Teixeira et al. [14] had a different study design, where prone acquisition was performed 60 min after injection, and supine imaging was performed after prone acquisition was completed. Nonetheless, they detected significantly more avid axillary LNs and better primary tumor multifocality in prone acquisition [14]. Consequently, it may be argued that the higher SUVmax is not necessarily due to the delayed acquisition of supine images, as there are studies with different designs showing opposing findings.

According to the current AJCC breast cancer staging, intramammary LNs do not change staging or management [11]. Hashem et al. [22] found that intramammary LNs may be associated with advanced pathological features, and this may lead to upstaging when axillary LNs are disease-free. In our study, we had two patients with FDG-avid intramammary LNs on prone, none of which were detected on supine. One patient already had axillary metastasis, while the other had no further lymphatic metastasis. Nassar et al. [23] discussed how intramammary LN metastasis is a poor prognostic marker; however, involved patients had axillary LN metastasis as well. This prevented them from assessing intramammary LN metastasis as an independent prognostic factor.

Axillary LNs were detected on prone in five more patients than on supine. The study conducted by Vidal-Sicart et al. had similar findings [24]. Even in patients where axillary LNs were detected in both positions, a higher number of FDG-avid LNs were detected on prone. Such a finding was detected in our study in 7/25 patients, while Vidal-Sicart et al. [24] had it in 1 patient out of 30. The significantly higher detection rate of axillary LNs in prone position can be explained by the wider axillary area visualized in this position [25]. In our study, the higher SUVmax detected on prone was statistically significant, similar to Teixeira et al. [14], who found a statistically significant higher avidity of axillary LNs on prone when compared to supine. In addition, anatomical mismatch of axillary LN metastasis was shown to occur more often on supine than on prone [13]. In our study, prone imaging had a higher sensitivity, specificity, and a lower false negative rate than that of supine. This may be explained by the muscle stretching in prone position when the patient’s arms are outstretched cranially and medially, allowing for a better view of LNs when compared to supine. This would also explain why more axillary LNs were picked up on prone in comparison to supine. There was a change in patient staging in a total of five patients. They were all upstaged and subsequently underwent chemotherapy in addition to surgery. Contrary to this finding, a recent study by van Loevezijn et al. [26] found that there was no significant change in the staging between prone and supine imaging. It is worthy of note that there were two observers in their study, with one upstaging 14 patients and the other having no difference in staging, although observers were blinded to other imaging findings.

Special consideration of patient history and clinical picture is required in the assessment of patient FDG PET/CT due to physiologic uptake in benign processes [27]. This is moreso true in the era of COVID-19 vaccination, where Skawran et al. [28] found that 54% of recently vaccinated patients had FDG-avid axillary LNs ipsilateral to the site of vaccination, and similar findings were elicited in several studies [29,30]. The economics and logistics of using prone positioning should also be considered: special mattresses would be required for prone acquisition, and more time would be needed for patient positioning, which may disrupt workflow in high-volume centers.

Our single center study was limited by the small sample size, with only 25 patients included. The second observed limitation is the higher SUVmax in prone imaging, which may be explained by the greater time interval from injection to acquisition time in the prone position inherent to this study’s design. A third limitation would be the inclusion of patients receiving axillary node dissection alongside patients undergoing targeted node biopsy. In the case of axillary node dissection, it is not possible to intraoperatively differentiate between normal nodes and nodes with FDG uptake if a gamma probe is not used. Accordingly, this results in ambiguity regarding the positivity of nodes on a one-to-one basis if a large heterogenous group of nodes is dissected during surgery. This introduces measurement bias in the study. Future studies comparing prone and supine FDG PET/CT to pathological standards in breast cancer staging should use targeted node biopsy and avoid dual-position imaging at one FDG injection to control for the injection-to-acquisition time interval. Larger, prospective randomized studies are needed before drawing stronger conclusions.

## 5. Conclusions

Prone FDG PET/CT acquisition is a promising technique in detecting primary breast lesions and metastatic LNs possibly missed in supine acquisition, which may lead to changes in patient staging and management. Owing to the design and small sample size of this study, further studies are required to better assess the actual accuracy of prone FDG PET/CT compared to supine acquisition in detecting lesions and metastasis.

## Figures and Tables

**Table 1:** Primary breast lesions detected on prone vs. supine.

Patient Number	Number of Primary Breast Lesions Detected on Prone	Highest SUVmax on Prone	Number of Primary Breast Lesions Detected on Supine	Highest SUVmax on Supine
1	2	21.11	2	15.40
2	2	5.97	1	4.96
3	1	3.30	0	-
4	1	2.30	0	-
5	1	4.20	1	3.30
6	1	3.96	0	-
7	1	7.15	1	5.20
8	1	5.90	1	3.80
9	1	12.30	1	7.30
10	1	5.50	1	5.50
11	3	4.11	1	3.90
12	1	19.90	1	18.30
13	2	4.70	1	4.40
14	Multiple	5.90	Multiple	4.20
15	1	8.40	1	7.70
16	1	6.00	1	4.70
17	1	32.90	1	30.20
18	1	7.10	1	5.50
19	1	7.50	1	5.90
20	1	13.80	1	4.30
21	1	9.56	1	6.80
22	1	7.40	1	3.00
23	1	20.30	1	9.90
24	1	11.50	1	4.80
25	1	5.30	1	2.10

**Table 2:** Lymph nodes detected on prone versus supine acquisition.

Patient Number	Intramammary Nodes (Prone; Supine)	Axillary Nodes (Prone; Supine)	Internal Mammary Nodes (Prone; Supine)	Supraclavicular Nodes (Prone; Supine)
1	0; 0	12; 4	0; 0	0; 0
2	0; 0	1; 1	0; 0	0; 0
3	0; 0	3; 1	0; 0	1; 0
4	0; 0	3; 0	2; 2	0; 0
5	0; 0	2; 0	0; 0	0; 0
6	1; 0	0; 0	0; 0	0; 0
7	0; 0	0; 0	0; 0	0; 0
8	0; 0	0; 0	0; 0	0; 0
9	0; 0	2; 0	0; 0	0; 0
10	0; 0	8; 3	1; 1	0; 0
11	0; 0	3; 2	0; 0	0; 0
12	0; 0	2; 1	0; 0	0; 0
13	0; 0	3; 4	0; 0	0; 0
14	1; 0	10; 3	0; 0	0; 0
15	0; 0	3; 0	0; 0	0; 0
16	0; 0	0; 0	0; 0	0; 0
17	0; 0	0; 0	0; 0	0; 0
18	0; 0	0; 0	0; 0	1; 1
19	0; 0	1; 0	0; 0	0; 0
20	0; 0	1; 0	0; 0	0; 0
21	0; 0	0; 0	0; 0	0; 0
22	0; 0	0; 0	0; 0	0; 0
23	0; 0	0; 0	0; 0	0; 0
24	0; 0	8; 6	0; 0	0; 0
25	0; 0	0; 0	0; 0	0; 0

## Data Availability

Data will be available by requesting from correspondence.
